# The Relative Claims of Operative and Chemical Dentistry

**Published:** 1864-05

**Authors:** O. P. Fitch


THE RELATIVE CLAIMS OF OPERATIVE AND CHEMICAL DENTISTRY.—*By C. P. Fitch, M. D.—Read before the Society of Dental Surgeons of the City of New York.*

The fact is quite apparent that each department of dental practice has its own specific claims, and demands certain qualifications on the part of those assuming the practice of either specialty. While this is apparent and its truthfulness readily conceded, it is not quite so clear in the minds of many gentlemen of our profession that the dental practitioner should manipulate in one or the other of these departments exclusively.

While we assume the affirmative of this statement, the conviction does not press heavily upon us that there exists any occasion for self-congratulation, or for instituting invidious comparisons, or for setting up claims of exclusiveness, by gentlemen officiating in either branch of practice, on account of superiority of intellection or of acquisition. Admitting that this, in a certain sense, may be so in an extremely limited number of cases, yet with many assuming this it will be found to be more fancied than real when subjected to catechestical tests, the *experimentum crusis* for all such assumptions. But, on the contrary, there is really an occasion for self-humiliation, and for the exercise of a spirit of uniform docility on account of the little that is known by the wisest among us, in comparison to the much that may and is destined to be known in the extended fields of dental research in reference to both its principles and practice. Indeed, the initiatory steps, in the curriculum of study, should commence with basal principles, and embrace, in its ever-widening circumference, all that can contribute to, or in any sense constitute, the requisite qualifications for practice in both the operative and mechanical departments. So far as qualifications, fraternal recognition, and equality of position are concerned, the profession should be, and really is, a unit; but in reference to practice it should be dual: its requirements seem to point to a division of labor and effort. There are reasons why this is so, growing out of the nature of the practice inseparably connected with either branch of the profession.

We turn our attention then, briefly, to a consideration of the claims or duties connected with these respective departments.

In a peculiar sense, operative dentistry is mechanical; but its efficient practice supposes prerequisite gifts, natural and acquired, which partake largely of philosophy and science; while mechanical dentistry, proper, requires for its highest development gifts and acquirements which entitle its representatives to eminent distinction and rank as machinists and artists. The operative department has to do with the conservative and radical treatment of oral diseases, the arrestation of dental decomposition, and the restoration of lost dental substance by the application of appropriate agencies to the natural dental organism; while mechanical dentistry calls upon its votaries to produce an artificial organ that will adequately substitute the loss of the natural, and the proper adjustment of the artistic product in its designed locality to subserve and simulate nature in utility and appearance. The specialist of the former should be conversant with nature's ever-varying normal and abnormal phases; should be familiar with her manifest and occult forces; should have at his command ample and specific remedial agencies and a knowledge of their legitimate application. The latter should understand all of nature, organic and inorganic, that he may make a suitable dental substitute whenever called to its performance. The one studies nature in order to protect and preserve her; the other studies nature that he may reproduce her.

The general acquisitions, requisite for efficiency in either department, do not materially differ; yet, in some specific sense they may, and doubtless do, differ. But the application of knowledge and gifts in the detail of manipulation *widely* differs. It is then in the line of practice, in operative and mechanical dentistry, that difference more particularly obtains, and that suitable natural gifts, and especially acquired fitness, are of the highest importance.

The practitioner of the former should be, in the highest sense, a mechanico-philosopher; able to originate, acquire with facility, and apply, in a practical sense, truths which constitute science; competent to suitably arrange and naturally group those truths which establish a fact or law; and apt in associating and comparing those general facts which, in the aggregate, are really the essence of philosophy. He should possess, in a marked degree, practical common sense; be endowed with quick perception, with nice discrimination, that natural relations and conventionalisms may be discerned and detected at a glance; but, in an important sense, he should possess more than ordinary manual dextrousness, that he may meet with promptness and perform with aptitude the heroic and finer manipulations incident to his calling. In order to command respect and maintain supremacy here, upon this the toil and recreation ground of his life's activities, it is quite important that he be self-possessed, firm but gentle, respecting, with becoming deference, the opinions of others, and yet maintaining his own views and convictions with earnestness and commendable zeal.

The practitioner of the latter specialty, whether he combines and presents in his complex being the highest endowments of philosophy and science or not, should be, in the fullest sense, a mechanico-artist. He should embody those qualities, and give practical demonstration of their existence, in all the products of his art, which would entitle him to confidence as a machinist and to eminence as an artist, in the reproduction of lost featural expression, there being present, as it were, but a few pieces of the skeleton of former expressional life from which to reconstruct the habitat of the spiritual entity, the soul's material representation. He should be familiar with nature's varied products, with chemical affinities, the laws of aggregation and segregation; should possess a nice appreciative sense of form, size, color; understanding the profound knowledge of temperaments, which determine, to a great extent, the character of the teeth to be supplied, and expert in the philosophy and practice of mechanics. Possessing such knowledge, and having such means and appliances at hand, he certainly should not fail, in any essential point, in meeting, to some considerable degree, the demands of his special calling. There are some, indeed, that ply their vocation in this department, that never have and never will originate anything. They live, fatten, and flourish upon the brain products of the few. They can not really be called mechanical dentists. The application of this term, so far as they are concerned, is certainly a misnomer. If these remarks are true in reference to some in the mechanical department, what must be said, if the truth were to be spoken, in reference to many in operative dentistry? Gentlemen, I can not do justice to this subject without committing offense, and as I wish, in all charity, to forbear giving pronouncement to remarks caustic or severe, I pass this thought, at least for the present, in silence.

But there is a practical phase of this subject which demands a moment's investigation. It is in a practical sense that the operative and mechanical departments should be kept quite separate and distinct: such a wide difference obtains, just here, that it seems almost indispensable to the attainment of eminence and high development in either, that exclusiveness be rigidly adhered to.

I do not use the term exclusiveness in its offensive sense, but simply to indicate a given line of practice. He who would reach distinction and desire to have his name associated with pleasant memories, or attain usefulness, in its highest and most extended sense, must concentrate his thought and energy in one or the other of these practical departments.

Many representative men in our profession, both in the past and present, could be referred to as fully sustaining and illustrating this position. In the minds of many, especially those having a small business, there exists good and sound reasons why these two branches should be united. But I can not conceive of an instance, unless it be found in the fact of a very limited practice, and in a locality in which it is practically impossible to secure additional services (and certainly this latter reason will not apply to our cities), where it would not be better for patient and operator to keep them quite distinct. If any reasons are valid, the two just alluded to seem to take the precedent; but even under these circumstances, it would be better for the operator, in reference to the former, simply to superintend the manipulations in the mechanical; and in relation to the latter, to make arrangements for procuring the requisite help as soon as possible.

Young men should be educated, true, in broad general principles, but, especially, with one or the other of these departments in view.

It is, indeed, quite inopportune to be called from the laboratory with hands immersed in sand and plaster; roughened and abraded by contact with its furniture and appliances, losing that nice sense of palpation so highly requisite for rendering delicate services; or with person besmeared and ill at ease from sundry contacts with that which is sure to leave its mark and remain somewhat indelible, but which, legitimately considered, is the insignia of commendable industry, of honorable and honest toil, and only in this instance unpleasantly noticeable because sadly out of place, or with the mental and physical nature intensified and heated to the soldering point—I say it is quite inopportune under these circumstances, to be called upon to meet demands of an operative character. Not only is the operator, at such times, incapacitated for rendering efficient service in that which requires precision and delicacy of touch in its elaborateness and detail, the highest states of finish, resembling nature in its results, which often is, and always should be, the embodiment of the operator's highest conceptions, but the delicate mucous surfaces must suffer and pay the penalty of such inconsiderate practice, and the feelings of the patient must be painfully outraged at the manifestation of such an utter want of physical fitness for the performance of so delicate and important an operation. Therefore I can see no occasion, whenever this subject is mooted (for really there none exists where it is properly understood), for the indulgence, on the part of any one, of unpleasant and improper feelings, or for an expression of bitter, reproachful language for supposed or claimed superiority of the operative to the mechanical; for he who sets up such a claim is decidedly fractional as it regards mental caliber and acquisition, and consequently his erroneous position has very little claim to either respect or notice. On the contrary, I can see many valid and sound reasons, founded in fact and requirement, why the practice of the operative and mechanical in dentistry should be exclusively pursued by different gentlemen of our profession.

In closing these few thoughts, gentlemen, I sincerely hope that a laudable ambition for superior excellence, respecting results will ever characterize, nerve, and stimulate every aspirant in either specialty, and that the fraternal feeling now so prevalent in this society, and cropping out so abundantly all over the land, will ever maintain the ascendancy, so that an honest expression of opinion, or even the contact of offensive contrast, either upon this or any other subject, may never tarnish our fair fame, neither destroy nor check the manifestation of reciprocal regard, or mar, in the slightest degree, the good time now enjoyed, and in the coming future sure.

NEW YORK, Jan., 1864.

